# Immunomodulatory insights of monoterpene glycosides in endometriosis: immune infiltration and target pathways analysis

**DOI:** 10.1186/s41065-024-00354-8

**Published:** 2025-01-03

**Authors:** Jin Zhan, Jiajie Wu

**Affiliations:** 1 Department of Gynaecology, Ningbo Municipal Hospital of Traditional Chinese Medicine, Affiliated Hospital of Zhejiang Chinese Medical University, No.819, Liyuan North Road, Haishu District, Ningbo, Zhejiang Province 315010 China; 2 Emergency Department, Ningbo Municipal Hospital of Traditional Chinese Medicine, Affiliated Hospital of Zhejiang Chinese Medical University, Ningbo, Zhejiang Province China

**Keywords:** Endometriosis, Paeonia lactiflora, Monoterpene glycosides, Immune cell infiltration, Immunomodulatory targets, Machine learning, CASP3 interaction

## Abstract

Endometriosis is a complex gynecological condition characterized by abnormal immune responses. This study aims to explore the immunomodulatory effects of monoterpene glycosides from Paeonia lactiflora on endometriosis. Using the ssGSEA algorithm, we assessed immune cell infiltration levels between normal and endometriosis groups. Key targets were identified through differential expression analysis of the GSE51981 dataset. Potential immunomodulatory targets of Paeonia lactiflora compounds were identified through Venn diagram analysis, followed by enrichment and machine learning analyses. A nomogram was developed for predicting endometriosis, while molecular docking explored compound-target interactions. Significant differences in immune cell infiltration were observed, with increased CD8 T cells, cytotoxic cells, and others in endometriosis. Differential expression analysis identified 43 potential targets. Enrichment analysis highlighted pathways involved in immune and inflammatory responses. Machine learning identified SSTR5, CASP3, FABP2, and SYK as critical targets, contributing to a nomogram that demonstrated good predictive performance for endometriosis risk. Molecular docking revealed strong interactions between Paeoniflorigenone and CASP3. Our findings suggest that monoterpene glycosides have therapeutic effects on endometriosis by modulating key immune-related targets and pathways, providing a basis for further investigation into Paeonia lactiflora’s potential as a treatment for this condition.

**Supplementary Information:**

The online version contains supplementary material available at 10.1186/s41065-024-00354-8.

## Introduction

Endometriosis is a prevalent gynecological disorder affecting approximately 10% of women of reproductive age, characterized by the presence of endometrial-like tissue outside the uterus, causing chronic pelvic pain and infertility [4, 5]. Despite extensive research, the precise etiology and pathogenesis of endometriosis remain elusive, largely due to its multifactorial nature involving genetic, hormonal, and immunological factors [17, 19]. Notably, the immune system plays a pivotal role in the development and progression of endometriosis, as evidenced by altered immune cell populations and cytokine profiles in affected individuals [6, 24, 44]. Understanding the immunological aspects of endometriosis is crucial for developing effective therapeutic strategies. Previous studies have highlighted the significance of immune cell infiltration in endometriosis, with increased levels of CD8 T cells and NK cells observed in ectopic lesions [15, 18, 31]. However, the exact mechanisms through which these immune cells contribute to disease pathology are not fully understood. Furthermore, current treatments for endometriosis, including hormonal therapies and surgical interventions, often provide only temporary relief and are associated with adverse side effects. This necessitates the exploration of novel therapeutic agents that can modulate the immune response and offer long-term benefits.

Paeonia lactiflora, a traditional Chinese medicinal herb, has garnered attention for its potential immunomodulatory properties [41]. Monoterpene glycosides, the active compounds derived from Paeonia lactiflora, have demonstrated anti-inflammatory and immunoregulatory effects in various disease models [26, 34, 43]. However, their specific role in endometriosis and the underlying molecular mechanisms remain largely unexplored. While previous research has established the immunomodulatory potential of these compounds, there remains a gap in understanding how they may specifically address the immune dysregulation observed in endometriosis. Recent studies from have begun to explore the intersection of machine learning approaches and endometriosis research, which could provide novel insights into this area [1, 12, 36]. Given the critical involvement of the immune system in endometriosis and the promising immunomodulatory effects of monoterpene glycosides from Paeonia lactiflora, this study aims to investigate the potential therapeutic effects of these compounds on endometriosis. By integrating bioinformatics and machine learning approaches, we aim to provide more precise insights into the immune-related pathways and key targets modulated by monoterpene glycosides, thereby advancing the current understanding of their mechanisms in the context of endometriosis.

The primary objective of this study is to explore the immunomodulatory effects of monoterpene glycosides from Paeonia lactiflora on endometriosis. This study employs a comprehensive approach combining bioinformatics and computational analyses. We utilize the ssGSEA algorithm to evaluate immune cell infiltration levels and differential expression analysis to identify key targets from the GSE51981 dataset. Potential immunomodulatory targets are identified through Venn diagram analysis, followed by enrichment and machine learning analyses. A nomogram is constructed to predict endometriosis risk, and molecular docking is performed to explore interactions between active compounds and key targets. Figure S1 presents the flow chart of the study. This research provides novel insights into the immunomodulatory mechanisms of monoterpene glycosides from Paeonia lactiflora in endometriosis. By identifying key immune-related targets and pathways, we offer a potential therapeutic strategy for managing endometriosis, addressing the limitations of current treatments, and paving the way for future clinical applications.

## Results

### Immune cell infiltration

**Fig. 1:**
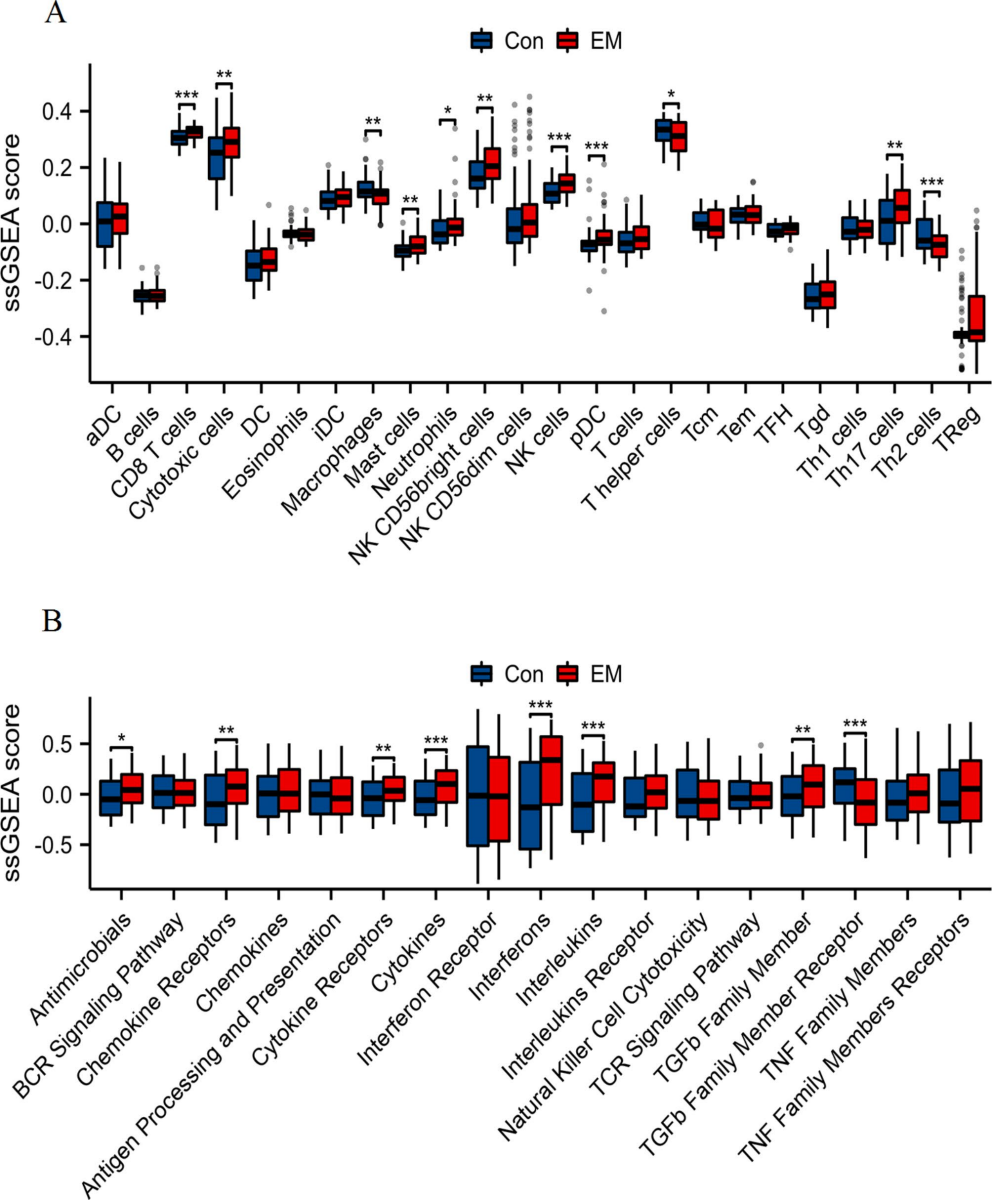
Comparison of immune cell infiltration levels and immune-related pathway scores. Immune cell infiltration levels and immune-related pathway scores in the Con and EM groups assessed using the ssGSEA algorithm. Statistical significance is indicated as follows: *p < 0.05, **p < 0.01, ***p < 0.001

The immune cell infiltration levels between the control (Con) and endometriosis (EM) groups were assessed using the ssGSEA algorithm (Fig. 1A). Our analysis revealed significant differences in several immune cell types. Notably, CD8 T cells, cytotoxic cells, mast cells, neutrophils, NK CD56bright cells, NK cells, pDC, and Th17 cells showed increased infiltration in the EM group compared to the normal group (*p < 0.05, **p < 0.01, ***p < 0.001). Conversely, a significant decrease was observed in macrophages, T helper cells and Th2 cells in the EM group compared to the normal group (*p < 0.05, **p < 0.01, ***p < 0.001). These findings suggest differential immune cell infiltration profiles between the normal and endometriosis groups. The ssGSEA algorithm was utilized to evaluate the immune-related pathway scores between the normal and endometriosis groups (Fig. 1B). Significant alterations were observed in several immune pathways. The antimicrobial pathway, chemokine receptors, cytokine receptors, cytokines, interferons, interleukins, and TGFb family member exhibited significantly higher scores in the EM group (*p < 0.05, **p < 0.01, ***p < 0.001). Conversely, TGFb family member receptor pathway showed significant decrease in score in the EM group (***p < 0.001). These results indicate that the immune-related pathways are more actively present in endometriosis patients, potentially contributing to the disease pathology.

Identification the potential immunomodulatory targets of monoterpene glycosides from Paeonia lactiflora in treating endometriosis.

**Fig. 2:**
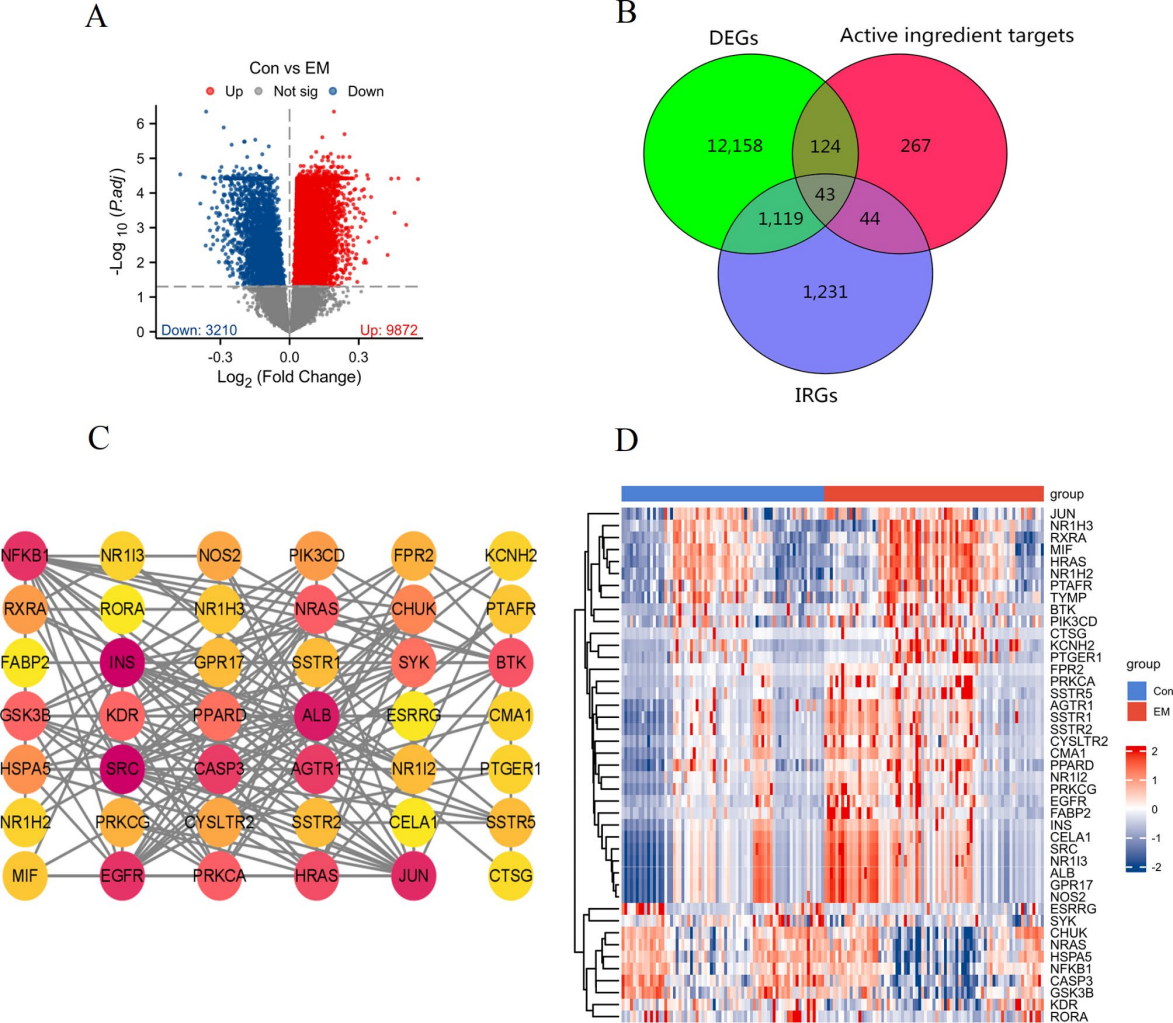
Exploration of potential immunomodulatory targets of monoterpene glycosides for the treatment of endometriosis. (**A**) Differential expression analysis based on the GSE51981 comparing Con and EM groups. (**B**) Venny diagram illustrating the intersection of DEGs, active ingredient targets from four monoterpene glycosides, and IRGs. A total of 43 common targets were identified. (**C**) PPI network analysis of the 43 common targets. (**D**) Heatmap displaying the expression profiles of the 43 common targets based on the GSE51981 dataset

Differential expression analysis between the Con and EM groups was conducted using the endometriosis-related dataset (GSE51981) (Fig. 2A). This analysis identified 9872 upregulated and 3210 downregulated differentially expressed genes (DEGs) in the EM group compared to the normal group. Four active compounds from Paeonia lactiflora (Paeoniflorigenone, Paeoniflorin, Benzoyl paeoniflorin, and Albiflorin) were selected for the study. Using three online databases (Super-PRED, PharmMapper, and SwissTargetPrediction), we identified 478 potential targets. Additionally, we retrieved 2437 immune-related genes (IRGs) from the ImmPort database. A Venny diagram analysis using the Venny tool intersected the DEGs, compound targets, and IRGs, resulting in the identification of 43 common targets. These 43 targets represent the potential immunomodulatory targets of the active compounds for the treatment of endometriosis (Fig. 2B). The PPI network displayed extensive interactions among the targets, suggesting key proteins that could be modulated by the active compounds in the context of endometriosis (Fig. 2C). The expression profiles of the 43 potential targets were visualized in a heatmap using the GSE51981 (Fig. 2D). The heatmap exhibits differential expression patterns of these targets between the normal and EM groups, further supporting their potential roles as therapeutic targets. These findings collectively propose that the monoterpene glycosides from Paeonia lactiflora might exert therapeutic effects against endometriosis through modulating these identified targets.

### Enrichment analysis of potential targets

**Fig. 3:**
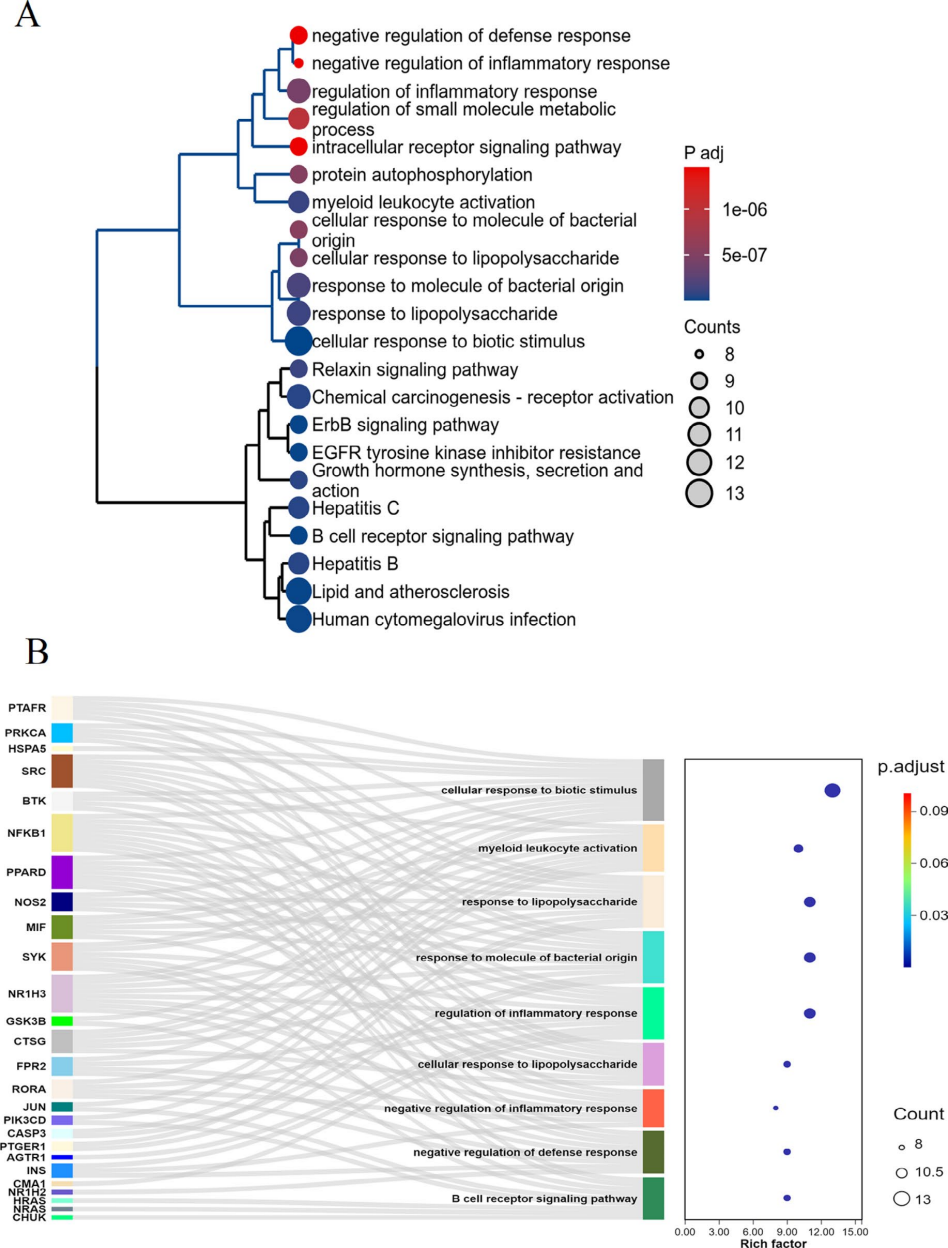
Enrichment analysis of the potential targets of monoterpene glycosides against endometriosis. (**A**) The clustering tree diagram displays significantly enriched pathways and biological processes, with the dot color representing the adjusted p-value and the dot size indicating the number of genes involved. (**B**) Visualization of the enrichment analysis results related to immune and inflammatory pathways. The network diagram shows the connection between the identified targets and enriched pathways. Each line represents the association between a target gene and an enriched pathway, with the color intensity and size of nodes corresponding to the adjusted p-value and the number of genes involved, respectively

The 43 potential targets identified for the monoterpene glycosides from Paeonia lactiflora against endometriosis were subjected to enrichment analysis. Figure 3A reveals the top enriched pathways and biological processes. The analysis indicates significant involvement in immune and inflammatory responses, including negative regulation of defense response, negative regulation of inflammatory response, and myeloid leukocyte activation. Other critical pathways include relaxin signaling pathway, erbB signaling pathway, EGFR tyrosine kinase inhibitor resistance, hepatitis C, etc. Figure 3B visualizes the enriched pathways particularly related to immune and inflammatory processes. The network diagram highlights the connection between the identified targets and relevant pathways such as myeloid leukocyte activation, response to lipopolysaccharides, cellular response to biotic stimulus, and the B cell receptor signaling pathway. Pathways like the negative regulation of inflammatory response and defense response further emphasize the significant role of these targets in modulating inflammatory processes in endometriosis. These comprehensive enrichment analyses demonstrate that the monoterpene glycosides from Paeonia lactiflora target key pathways involved in immune regulation and inflammation, providing potential therapeutic avenues for managing endometriosis through these mechanisms.

### Identification of key anti-endometriosis targets using machine learning

**Fig. 4:**
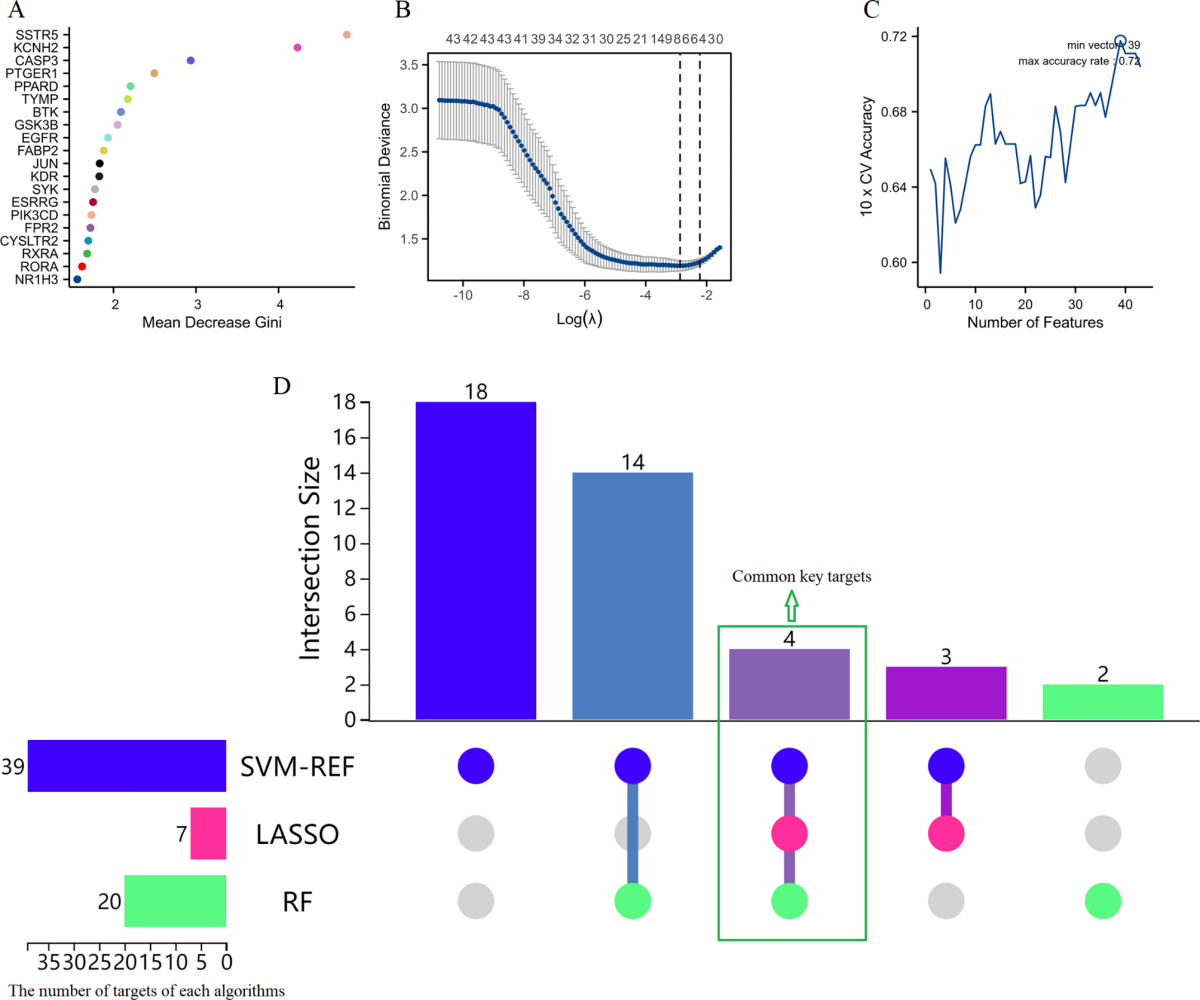
Identification of critical targets for treating endometriosis through the utilization of machine learning techniques. (**A**) Feature importance ranking of the 43 potential targets using the RF algorithm. (**B**) LASSO regression analysis results for the 43 potential targets. (**C**) SVM-REF analysis identifying the number of features that maximize the 10-fold cross-validation accuracy. (**D**) UpSet diagram integrating the results from RF, LASSO, and SVM-REF analyses

A feature importance analysis was conducted using the Random Forest (RF) algorithm, ranking the importance of the 43 targets based on the Mean Decrease Gini index (Fig. 4A). SSTR5, KCNH2, CASP3, PTGER1, PPARD, and TYMP emerged as the top-ranked targets, indicating their potential significance in the treatment of endometriosis. The LASSO regression analysis identified optimal targets by minimizing the binomial deviance (Fig. 4B). The analysis selected 7 key targets with a lambda value optimized at the point of minimum deviance. Feature selection using SVM-REF revealed the number of features that maximized the 10-fold cross-validation (CV) accuracy (Fig. 4C). The model achieved the highest accuracy with 39 features, underscoring their relevance in distinguishing between normal and endometriosis conditions. Integrating the results from RF, LASSO, and SVM-REF, a UpSet diagram was constructed to identify the overlapping key targets (Fig. 4D). Four common key targets (SSTR5, CASP3, FABP2, and SYK) were identified across all three machine learning methods, suggesting their crucial role as therapeutic targets for the anti-endometriosis effects of monoterpene glycosides from Paeonia lactiflora.

### Construction and evaluation of a nomogram for predicting endometriosis risk

**Fig. 5:**
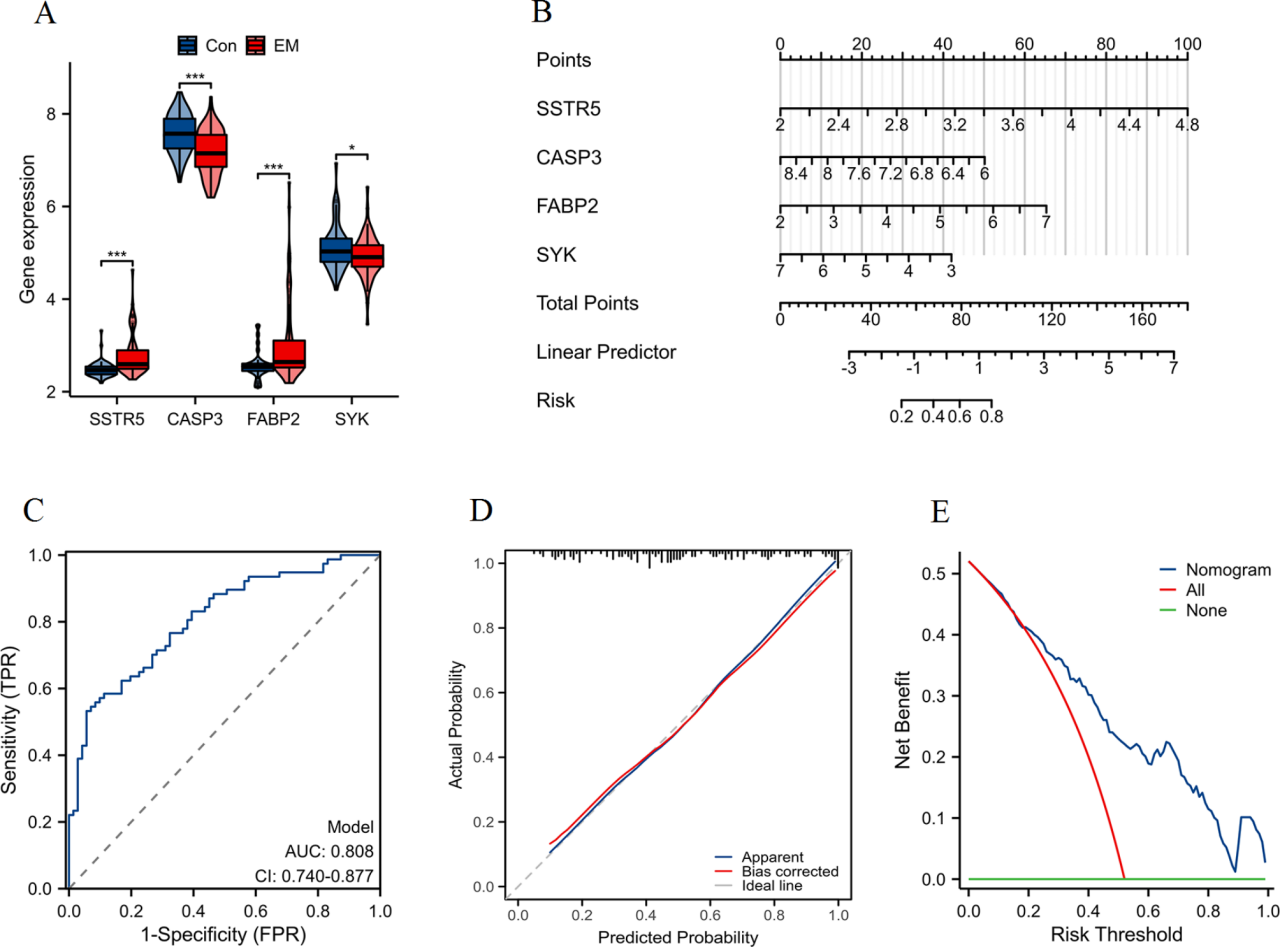
Construction and evaluation of a nomogram. (**A**) Gene expression profiles of the four key targets (SSTR5, CASP3, FABP2, and SYK) in Con and EM groups. Statistical significance is indicated as follows: *p < 0.05, ***p < 0.001. (**B**) Nomogram constructed based on the expression levels of the four key targets to predict the risk of endometriosis. (**C**) ROC curve evaluating the discriminative ability of the nomogram. (**D**) Calibration curve assessing the agreement between predicted probabilities and actual outcomes. (**E**) Decision curve analysis illustrating the net benefit of using the nomogram across a range of risk thresholds

The expression profiles of the four key anti-endometriosis targets (SSTR5, CASP3, FABP2, and SYK) were analyzed. Figure 5A illustrates significant upregulation of SSTR5 and FABP2, and downregulation of CASP3 and SYK in EM patients compared to the Con group. Based on the gene expression levels of the four identified targets, a nomogram was constructed to predict the risk of EM (Fig. 5B and Table S1). The nomogram assigns scores to each gene expression level, and the total score corresponds to a predicted risk of EM. The ROC curve (Fig. 5C) demonstrates the model’s discriminative ability, with an Area Under the Curve (AUC) of 0.808, indicating good predictive performance. The calibration plot shows that the nomogram predictions closely match the observed probabilities, indicating good calibration (Fig. 5D). The nomogram yields a higher net benefit across a range of risk thresholds compared to treating all or none, emphasizing its potential clinical value (Fig. 5E). A higher net benefit indicates that the model reduces the number of false positives and false negatives, thus optimizing patient management by improving the accuracy of EM risk predictions. Clinically, this means that the nomogram can help identify high-risk patients more accurately, enabling more targeted interventions and reducing unnecessary treatments for those at low risk.

### Correlation analysis of key anti-endometriosis targets with immune cell infiltration

**Fig. 6:**
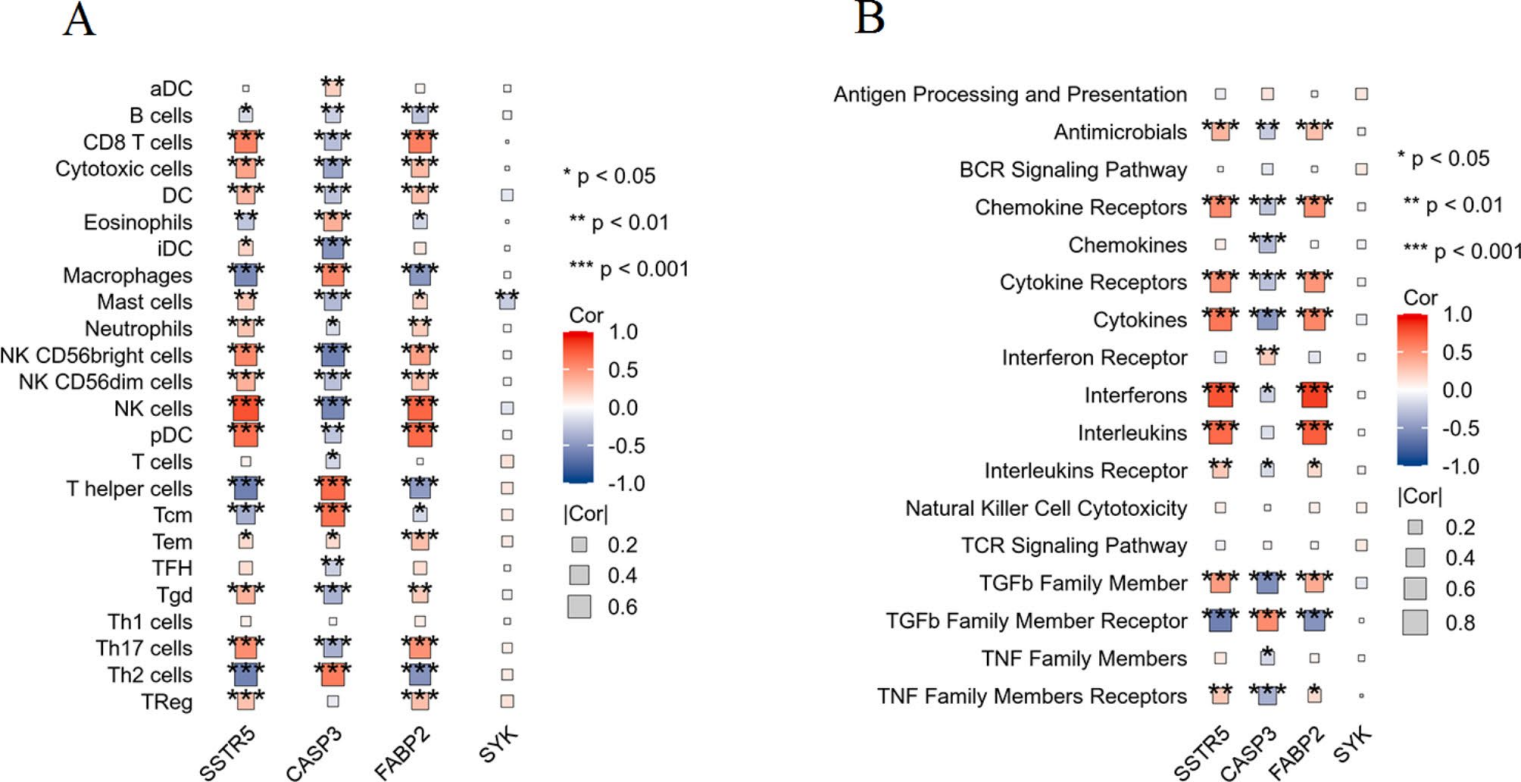
Correlation analysis of key anti-endometriosis targets with immune cell infiltration and immune pathways. (**A**) Correlation heatmap of the four key anti-endometriosis targets with immune cell infiltration. (**B**) Correlation heatmap of the four key targets with immune-related pathways

The correlation between gene expression of the four key anti-endometriosis targets (SSTR5, CASP3, FABP2, and SYK) and immune cell infiltration was analyzed. Figure 6A shows the correlation heatmap for various immune cell types. Notably, CASP3 expression exhibited significant negative correlations with several immune cell types including CD8 T cells, cytotoxic cells, DC, iDC, NK CD56bright cells, NKCD56dim cells, NK cells, and Th17 cells (*p* < 0.001). In contrast, SSTR5 and FABP2 expression showed significant positive correlations with several immune cell types, such as D8 T cells, cytotoxic cells, DC, NK CD56bright cells, NKCD56dim cells, NK cells, and Th17 cells (*p* < 0.001). Figure 6B illustrates the correlation heatmap between the gene expression of the four key targets and various immune-related pathways. CASP3 showed strong negative correlations with chemokine receptors, chemokines, cytokine receptors, cytokines, TGFb family member, and TNF family members receptors pathways (*p* < 0.001). SSTR5 and FABP2 exhibited significant positive correlations with several immune pathways, including the antimicrobials, chemokine receptors, cytokine receptors, cytokines, interferons, interleukins, and TGFb family member pathways (*p* < 0.001). These correlation analyses underscore the significant involvement of the key anti-endometriosis targets (SSTR5, CASP3, FABP2, and SYK) in modulating immune cell infiltration and immune-related pathways. This highlights the potential immunomodulatory mechanisms by which the monoterpene glycosides from Paeonia lactiflora exert their therapeutic effects against EM.

### Molecular docking analysis of key targets with active compounds

**Fig. 7:**
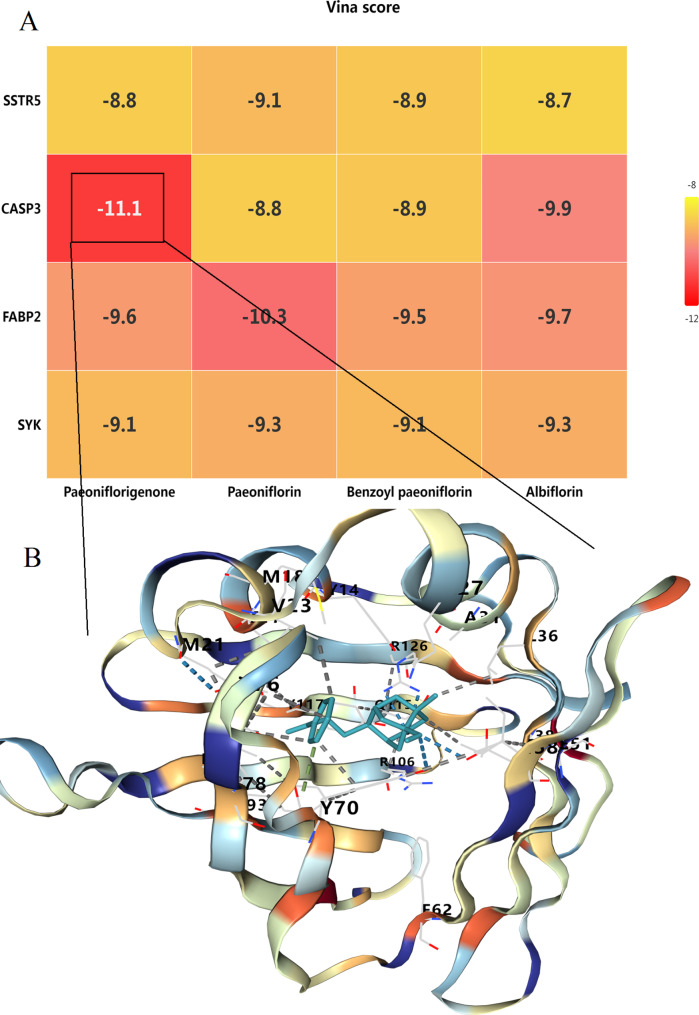
Molecular docking analysis of key anti-endometriosis targets with monoterpene Glycosides. (**A**) Heatmap of Vina docking scores showing the binding affinities between the four key anti-endometriosis targets and the four monoterpene glycosides from Paeonia lactiflora. (**B**) Molecular docking visualization of CASP3 with Paeoniflorigenone

Figure 7A displays the docking scores represented as Vina scores. Among the four targets, CASP3 showed the highest binding affinity with Paeoniflorigenone, with a Vina score of -11.1, indicating a strong interaction. The other compounds also exhibited notable binding affinities with the targets; Paeoniflorin displayed significant binding with FABP2 (Vina score: -10.3) and SYK (Vina score: -9.3). The varying Vina scores across different compounds and targets highlight the differential binding propensities, suggesting specific interactions relevant to the therapeutic effects of the compounds. Figure 7B provides a detailed visualization of the molecular docking between CASP3 and Paeoniflorigenone. The docking illustration shows the binding site and interactions, including hydrogen bonds and hydrophobic interactions. Key residues involved in the interaction include R126, M21, Y70, etc. These interactions suggest potential mechanisms thorugh which Paeoniflorigenone could modulate CASP3 activity, thereby exerting therapeutic effects against endometriosis. These docking results support the hypothesis that monoterpene glycosides from Paeonia lactiflora can effectively interact with key targets involved in endometriosis, potentially modulating their activity and contributing to the therapeutic effects observed.

## Discussion

Paeonia lactiflora, commonly known as white peony, has long been used in traditional Chinese medicine for its wide range of pharmacological effects, including anti-inflammatory, analgesic, and immunomodulatory properties [26, 40]. Recent studies have suggested that its active constituents, including monoterpene glycosides, may hold therapeutic potential for gynecological disorders such as endometriosis [25, 42]. Endometriosis is characterized by ectopic endometrial tissue growth, often accompanied by dysregulated immune responses and chronic inflammation [16, 27, 33]. Our study provides novel insights into the immunomodulatory effects of monoterpene glycosides from Paeonia lactiflora on endometriosis, highlighting their potential as a therapeutic agent.

In this study, we observed significant alterations in immune cell infiltration between normal and endometriosis tissues, with an increase in CD8 T cells and cytotoxic cells in the latter. These findings are consistent with previous reports that suggest a role for immune dysregulation in the pathogenesis of endometriosis [9, 10, 30]. Our differential expression analysis identified 43 potential targets, and enrichment analysis revealed pathways predominantly involved in immune and inflammatory responses. Notably, machine learning approaches identified key targets such as SSTR5, CASP3, FABP2, and SYK, which are implicated in immune modulation and apoptosis. Further exploration of the clinical implications of these targets, raises questions about their feasibility for translation into clinical applications. For instance, exploring the development of targeted therapies or biomarkers based on these targets could provide valuable insights into future treatment strategies for endometriosis. The identification of CASP3 as a key target is particularly noteworthy, as caspase-3 is a critical executor of apoptosis, and its dysregulation has been linked to the survival of ectopic endometrial cells [23]. Our molecular docking analysis revealed strong interactions between Paeoniflorigenone, a monoterpene glycoside, and CASP3, suggesting that Paeonia lactiflora may induce apoptosis in ectopic endometrial cells by modulating caspase-3 activity. This aligns with prior research indicating that Paeonia lactiflora exerts anti-apoptotic effects in various cell types [20, 21, 38, 39]. However, it is essential to acknowledge potential side effects and limitations of herbal therapies, as they may vary among individuals. A balanced perspective on these aspects will enhance the understanding of Paeonia lactiflora’s therapeutic potential. The construction of a nomogram based on the identified targets provided a reliable tool for predicting endometriosis risk, with an AUC of 0.808, indicating good predictive performance. This model’s predictive capacity adds to the growing body of evidence supporting the role of immune-related targets in the etiology and progression of endometriosis [45].

Our findings contribute to the current understanding of endometriosis by elucidating the immunomodulatory mechanisms of monoterpene glycosides from Paeonia lactiflora. The integration of immune infiltration analysis with machine learning and molecular docking represents a novel approach in endometriosis research, offering a comprehensive understanding of how these compounds may exert therapeutic effects. Nonetheless, it is important to consider potential limitations of our computational findings, such as biases inherent in the GSE51981 dataset and concerns regarding the predictive accuracy of machine learning models. Addressing these limitations will enhance the robustness of our conclusions. While our study provides compelling evidence for the potential of Paeonia lactiflora in endometriosis therapy, further experimental validation and clinical studies are warranted to fully elucidate its therapeutic efficacy and underlying mechanisms.

## Conclusions

In conclusion, our study identifies monoterpene glycosides from Paeonia lactiflora as promising candidates for the treatment of endometriosis, potentially through the modulation of immune-related targets and pathways. To further validate these findings, we recommend conducting experimental studies or clinical trials to test the therapeutic efficacy of Paeonia lactiflora in endometriosis. These findings not only enhance our understanding of the immunopathogenesis of endometriosis but also pave the way for the development of novel therapeutic strategies based on traditional medicine.

## Methods

### Active ingredient target screening

To identify potential active ingredients of Paeonia lactiflora for the treatment of endometriosis, we employed a systematic screening approach using the Traditional Chinese Medicine Systems Pharmacology Database and Analysis Platform (TCMSP) [28]. The screening criteria were set to an oral bioavailability (OB) ≥ 30% and drug-likeness (DL) ≥ 0.1. Based on these parameters and extensive literature review, four compounds from Paeonia lactiflora were selected: Paeoniflorigenone, Paeoniflorin, Benzoyl Paeoniflorin, and Albiflorin. The canonical SMILES of these compounds were retrieved from the PubChem database. Using these SMILES, we predicted the potential targets of the compounds through three distinct databases: Super-PRED, PharmMapper, and SwissTargetPrediction [7, 11, 35]. After obtaining the predicted targets from each database, duplicate targets were removed to consolidate the results.

### Disease target screening

We conducted a search for datasets related to endometriosis in the GEO database, utilizing “endometriosis” as the search term. The inclusion criteria for the datasets were as follows: (1) the organism must be Homo sapiens; (2) the sample size for both the disease and control cohorts should be no less than 40. Following our screening process, the GSE51981 dataset, which comprises 71 Con samples and 78 EM samples, fulfilled the criteria. To identify differentially expressed genes (DEGs) associated with endometriosis, we utilized the publicly available dataset GSE51981 from the Gene Expression Omnibus (GEO) database. The raw matrix files of the GSE51981 dataset were extracted and preprocessed using the affy package (v.3.8). This step involved background correction, normalization, and summarization of probe-level data to ensure accurate and reliable expression measurements. This dataset was annotated using the GPL570 platform annotation file to convert probe IDs to gene symbols. For cases where multiple probes corresponded to a single gene symbol, the average expression value of the probes was used to represent the gene’s expression level. The limma package (v.3.22.7) was employed to perform differential expression analysis between the normal and disease groups within the dataset. DEGs were identified based on an adjusted p-value threshold of < 0.05. The ggplot2 package (v.3.3.6) was used to visualize the results of the differential expression analysis.

### Monoterpene glycosides anti-endometriosis target screening

Immune-related genes (IRGs) were retrieved from the ImmPort database (http://www.immport.org). This dataset provided a comprehensive list of genes involved in immune responses [2]. DEGs identified from the GSE51981 dataset, IRGs from the ImmPort database, and predicted targets of the Monoterpene glycosides (Paeoniflorigenone, Paeoniflorin, Benzoyl Paeoniflorin, Albiflorin) were intersected using the Venny tool. The intersected genes were considered as the Monoterpene glycosides anti-endometriosis targets.

### Construction of protein-protein Interaction (PPI) network

The common targets obtained from the intersection analysis were submitted to the STRING database (https://string-db.org) [32]. The interaction data for the common targets were extracted from STRING with a high confidence score (interaction score > 0.7) to ensure the reliability of the interactions included in the network. The extracted PPI network data were imported into Cytoscape software (version 3.7.2) for visualization and further analysis.

### Enrichment analysis of monoterpene glycosides anti-endometriosis targets

To explore the biological functions and pathways associated with the common targets of monoterpene glycosides in the treatment of endometriosis, we conducted Gene Ontology (GO) functional and Kyoto Encyclopedia of Genes and Genomes (KEGG) pathway enrichment analyses. The common targets identified were subjected to enrichment analysis using ClusterProfiler package (version 2.2.7). For both GO and KEGG enrichment analyses, a significance threshold of *p* < 0.05 was applied to ensure the identification of statistically significant biological functions and pathways.

### Machine learning identification of core monoterpene glycosides anti-endometriosis targets

To identify the core targets of monoterpene glycosides for the treatment of endometriosis, we utilized three distinct machine learning algorithms based on the gene expression profiles of the common targets from the GSE51981 dataset. The selection of these specific machine learning models was based on their proven effectiveness in handling high-dimensional biological data and their ability to provide interpretable results. The Random Forest algorithm was employed using the randomForest package in R. The importance of each gene was ranked based on the mean decrease in accuracy, which allows for robust feature selection and helps in understanding the contribution of each gene to the model’s predictive power [14]. LASSO regression was conducted using the glmnet package in R. This method applies a penalty to the absolute size of the regression coefficients, effectively shrinking some coefficients to zero and thereby performing variable selection and regularization. LASSO is particularly useful in scenarios with many predictors, as it helps to prevent overfitting and enhances model interpretability [22]. The optimal lambda value was determined through cross-validation to identify significant predictors among the common targets. SVM-RFE was implemented using the e1071 package in R. This method ranks features by recursively eliminating the least significant ones and re-fitting the model until the optimal set of features is achieved. SVM-RFE is advantageous for its ability to handle non-linear relationships and its effectiveness in identifying the most relevant features in complex datasets [29]. The results from the RF, LASSO, and SVM-RFE analyses were integrated using the Venny tool. Common targets identified by all three methods were considered core Monoterpene glycosides anti-endometriosis targets [8, 37].

### Nomogram construction and evaluation

Using the rms package in R, we integrated the core target genes to construct a nomogram. The predictive ability of the nomogram for endometriosis was assessed through Receiver Operating Characteristic (ROC) curve analysis. To assess the accuracy of the nomogram, calibration curve analysis was performed. Decision Curve Analysis was conducted to evaluate the clinical utility and net benefit of the nomogram across different threshold probabilities.

### Immune characterization analysis

Gene sets used for evaluating the enrichment levels of various immune cell subsets were obtained from a previous study [3]. Additionally, gene sets related to immune response pathways were downloaded from the ImmPort database (http://www.immport.org) [2]. The enrichment levels of immune cell subsets and immune response pathways in endometriosis and normal samples were assessed using single-sample gene set enrichment analysis (ssGSEA) [13]. The results of the ssGSEA were visualized using the ggplot2 package. To explore the associations between core target genes and immune cell components as well as immune response pathways, pearson correlation analysis was performed. The correlation coefficients were calculated to determine the strength of the relationships. The results of the correlation analysis were also visualized using the ggplot2 package.

### Molecular docking analysis

To investigate the interaction between small molecule compounds and core target proteins associated with endometriosis, we conducted a molecular docking analysis. Small molecule compounds were obtained from the PubChem database in the form of Structure Data Files (SDF). These files were processed and converted into the PDBQT file for docking analysis using AutoDock Tools (version 1.5.6). The three-dimensional structures of the core target proteins were downloaded from the Protein Data Bank (PDB) (https://www.rcsb.org). These structures were prepared for docking by removing water molecules, adding hydrogen atoms, and assigning appropriate charges using AutoDock Tools. Molecular docking was performed using AutoDock to predict the binding affinity and interaction modes between the small molecule compounds and core target proteins. The docking results were analyzed to identify the best binding poses based on the binding energy scores. The interactions between the ligands and the target proteins were visualized using PyMOL software (version 1.0.0).

## Electronic supplementary material

Below is the link to the electronic supplementary material.

Supplementary Material 1

## Data Availability

The data used in our study are available from the GEO (https://www.ncbi.nlm.nih.gov/geo/) database.

## Abbreviations

TCMSP: Traditional Chinese Medicine Systems Pharmacology Database and Analysis Platform
OB: Oral bioavailability
DL: Drug-likeness
DEGs: Differentially expressed genes
GEO: Gene Expression Omnibus
IRGs: Immune-related genes
PPI: Protein-Protein Interaction
GO: Gene Ontology
KEGG: Kyoto Encyclopedia of Genes and Genomes
ROC: Receiver Operating Characteristic
ssGSEA: Single-sample gene set enrichment analysis
SDF: Structure Data Files
PDB: Protein Data Bank
Con: Control
EM: Endometriosis
