# Supplementary material for: Immunomodulatory insights of monoterpene glycosides in endometriosis: immune infiltration and target pathways analysis
Source: Hereditas. 2025 Jan 3;162:1. doi: 10.1186/s41065-024-00354-8 (PMC11697917; doi:10.1186/s41065-024-00354-8)
Supplement: Supplementary file 1 — Supplementary Material 1 [file 41065_2024_354_MOESM1_ESM.docx]

Supplementary figures


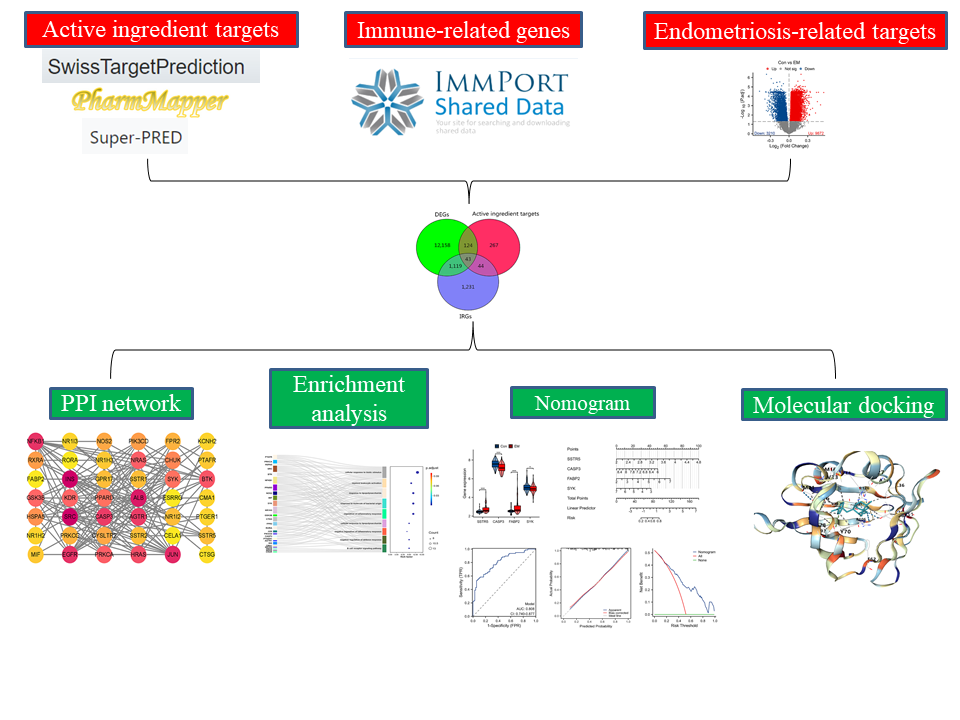


Figure S1 Flowchart of the study.

Supplementary tables

Table S1 Predictive value and correlation coefficient of the nomogram.

| Groups | SSTR5 | CASP3 | FABP2 | SYK | linear_predictors | risk score |
| --- | --- | --- | --- | --- | --- | --- |
| Con | 2.430193 | 8.28718 | 2.481574 | 4.866116 | -1.625518069 | 0.164445266 |
| Con | 2.341987 | 7.891851 | 2.372855 | 4.985564 | -1.499867386 | 0.182445304 |
| Con | 2.381351 | 8.011858 | 2.448223 | 4.988619 | -1.496910574 | 0.182886753 |
| Con | 2.371488 | 7.941632 | 2.109406 | 5.152873 | -1.85536632 | 0.13524406 |
| Con | 2.405872 | 7.496724 | 2.444124 | 4.803912 | -0.612317308 | 0.35153077 |
| Con | 2.301387 | 7.571377 | 2.395256 | 4.632628 | -0.891733907 | 0.29075214 |
| Con | 2.29035 | 8.028385 | 2.147152 | 4.81587 | -1.893015951 | 0.130900975 |
| Con | 2.384436 | 8.092233 | 2.120923 | 5.213472 | -2.059383546 | 0.113107654 |
| Con | 2.408484 | 7.714092 | 2.459405 | 4.804815 | -0.884696815 | 0.29220543 |
| Con | 2.352874 | 7.566131 | 2.092849 | 5.193671 | -1.441928131 | 0.191246944 |
| Con | 2.422483 | 8.101183 | 2.454412 | 4.910759 | -1.452087216 | 0.189680549 |
| Con | 2.359017 | 7.876029 | 2.098313 | 4.863645 | -1.596834045 | 0.168424566 |
| Con | 2.617129 | 7.825446 | 2.35263 | 5.338028 | -1.002393813 | 0.26847103 |
| Con | 2.346579 | 8.193826 | 2.412331 | 5.238988 | -2.043654367 | 0.114695144 |
| Con | 2.387578 | 8.109086 | 2.419047 | 5.722864 | -2.175492737 | 0.101972938 |
| Con | 2.415116 | 7.157628 | 2.559014 | 4.991853 | -0.166533232 | 0.458462645 |
| Con | 2.33 | 7.119317 | 2.449576 | 5.033208 | -0.45653922 | 0.387807138 |
| Con | 2.414822 | 7.252213 | 2.465878 | 4.442415 | 0.022645513 | 0.505661136 |
| Con | 2.563668 | 7.48288 | 2.617871 | 4.885716 | -0.103002368 | 0.47427215 |
| Con | 2.554703 | 7.227543 | 3.210357 | 5.029328 | 0.651779459 | 0.657411348 |
| Con | 2.541187 | 7.191046 | 2.616319 | 5.212993 | -0.007440433 | 0.4981399 |
| Con | 2.354506 | 7.522819 | 2.452996 | 4.599862 | -0.617877476 | 0.35026434 |
| Con | 2.507005 | 6.940455 | 2.611495 | 4.746304 | 0.581151603 | 0.641332347 |
| Con | 2.520356 | 6.78395 | 2.573643 | 5.562266 | 0.193632629 | 0.548257473 |
| Con | 2.562945 | 7.480769 | 3.044795 | 5.173481 | 0.075971437 | 0.51898373 |
| Con | 2.653429 | 6.904204 | 2.589541 | 4.413691 | 1.217292748 | 0.771586772 |
| Con | 2.532285 | 7.3965 | 2.585824 | 4.775404 | -0.013468188 | 0.496633004 |
| Con | 2.592639 | 7.228048 | 2.615789 | 4.682451 | 0.458249096 | 0.612598731 |
| Con | 2.478701 | 7.322381 | 2.531107 | 5.148777 | -0.369914867 | 0.408561593 |
| Con | 2.512359 | 7.254534 | 2.565477 | 5.349451 | -0.310486474 | 0.422996001 |
| Con | 2.562258 | 7.36356 | 2.596029 | 5.085688 | -0.11224243 | 0.471968815 |
| Con | 2.548641 | 7.529626 | 2.602526 | 5.130057 | -0.395815749 | 0.402318065 |
| Con | 3.319469 | 7.461302 | 3.439377 | 5.368551 | 2.200089423 | 0.900257541 |
| Con | 2.435566 | 6.975715 | 2.467668 | 4.779539 | 0.200949114 | 0.550068908 |
| Con | 2.384777 | 7.397677 | 2.526868 | 5.373142 | -0.872623951 | 0.294708606 |
| Con | 2.501925 | 7.619699 | 2.554822 | 5.213748 | -0.737643881 | 0.323519577 |
| Con | 2.99855 | 7.182863 | 3.3977 | 4.884382 | 2.092177736 | 0.890140568 |
| Con | 2.543384 | 7.423673 | 2.576906 | 5.256963 | -0.382599776 | 0.405500016 |
| Con | 2.490493 | 7.191832 | 2.543149 | 5.046529 | -0.079427393 | 0.480153584 |
| Con | 2.625043 | 7.090788 | 2.543232 | 4.676667 | 0.661535501 | 0.659605233 |
| Con | 2.505407 | 7.579065 | 2.538429 | 5.008069 | -0.538898559 | 0.368443843 |
| Con | 2.466868 | 7.1833 | 2.609185 | 5.171921 | -0.158338771 | 0.460497803 |
| Con | 2.487259 | 6.771707 | 2.539846 | 4.999373 | 0.508600742 | 0.624478399 |
| Con | 2.485426 | 6.528306 | 2.888075 | 5.029296 | 1.126024358 | 0.755104462 |
| Con | 2.516691 | 6.902811 | 2.559615 | 4.993026 | 0.428241065 | 0.605453573 |
| Con | 2.472211 | 7.063404 | 2.916062 | 4.918945 | 0.480193783 | 0.617793633 |
| Con | 2.61726 | 7.639305 | 2.672596 | 6.042731 | -0.976133037 | 0.273659747 |
| Con | 2.628474 | 7.376062 | 2.684047 | 5.038854 | 0.149960499 | 0.537420025 |
| Con | 2.64064 | 7.707547 | 3.260639 | 4.564706 | 0.605921823 | 0.647009955 |
| Con | 2.62873 | 7.896798 | 2.684308 | 4.787928 | -0.365497864 | 0.409629343 |
| Con | 2.511312 | 7.604087 | 2.694737 | 5.510923 | -0.783333712 | 0.31360184 |
| Con | 2.630035 | 7.676593 | 3.073404 | 5.494953 | -0.22935405 | 0.442911522 |
| Con | 2.599735 | 7.588153 | 3.441155 | 5.817506 | -0.087192153 | 0.478215761 |
| Con | 2.361081 | 7.967965 | 2.421504 | 4.940972 | -1.477806054 | 0.185759031 |
| Con | 2.453783 | 8.143599 | 2.505662 | 5.207534 | -1.601616673 | 0.167755784 |
| Con | 2.186132 | 7.751873 | 2.509319 | 5.839706 | -2.199807087 | 0.099767814 |
| Con | 2.338094 | 7.536724 | 2.191331 | 6.100389 | -2.012565486 | 0.117889926 |
| Con | 2.432884 | 7.918447 | 2.50673 | 4.474036 | -0.813727367 | 0.307096786 |
| Con | 2.311303 | 7.933321 | 2.487511 | 6.929772 | -2.949195727 | 0.049774538 |
| Con | 2.415909 | 7.317047 | 2.559854 | 4.770974 | -0.216533219 | 0.446077219 |
| Con | 2.466392 | 7.782273 | 2.518538 | 4.491369 | -0.549316048 | 0.366023106 |
| Con | 2.498748 | 8.329783 | 2.551578 | 5.364593 | -1.813058379 | 0.140268899 |
| Con | 2.393761 | 7.456123 | 2.20937 | 4.614075 | -0.662611895 | 0.34015313 |
| Con | 2.462458 | 7.871082 | 2.51173 | 5.402737 | -1.351019877 | 0.205703684 |
| Con | 2.471104 | 7.727696 | 2.477603 | 4.537366 | -0.535119423 | 0.369323658 |
| Con | 2.533823 | 8.184878 | 2.595129 | 4.198282 | -0.638680026 | 0.345544982 |
| Con | 2.370983 | 7.724919 | 2.289271 | 6.115205 | -2.10541717 | 0.108571415 |
| Con | 2.229748 | 8.46699 | 2.559782 | 5.275479 | -2.593896535 | 0.069532263 |
| Con | 2.345714 | 7.816437 | 2.504381 | 4.968511 | -1.257140872 | 0.221466468 |
| Con | 2.357788 | 7.99132 | 2.210374 | 6.179702 | -2.615173369 | 0.068168249 |
| Con | 2.319856 | 8.070592 | 2.266697 | 5.918001 | -2.573466716 | 0.070865703 |
| EM | 2.612088 | 7.194651 | 2.667314 | 4.704897 | 0.581961804 | 0.641518692 |
| EM | 3.019567 | 7.545964 | 4.807866 | 5.015308 | 2.843491973 | 0.944981298 |
| EM | 3.466555 | 6.87715 | 4.986609 | 4.214837 | 5.601949173 | 0.996322908 |
| EM | 2.652196 | 7.634365 | 5.985495 | 4.839437 | 3.010753323 | 0.953057569 |
| EM | 2.593636 | 7.061325 | 3.368057 | 4.374859 | 1.593978725 | 0.831175146 |
| EM | 2.79214 | 7.699335 | 3.195454 | 5.007727 | 0.610551675 | 0.648066636 |
| EM | 3.42545 | 7.627932 | 6.514319 | 3.918489 | 6.097092467 | 0.99775565 |
| EM | 2.757663 | 7.643215 | 4.398698 | 5.522174 | 1.318572725 | 0.788944147 |
| EM | 2.578651 | 7.526611 | 4.195618 | 5.500297 | 0.861294545 | 0.702931051 |
| EM | 2.597425 | 7.724488 | 2.652341 | 5.402358 | -0.690137275 | 0.334002536 |
| EM | 2.583639 | 7.536555 | 2.96956 | 5.220646 | -0.050449565 | 0.487390283 |
| EM | 2.587876 | 7.464843 | 3.519843 | 5.081879 | 0.658481112 | 0.658919109 |
| EM | 2.65081 | 7.713328 | 4.88088 | 4.812992 | 1.915730799 | 0.871661606 |
| EM | 2.590628 | 7.561572 | 3.028721 | 4.637287 | 0.413671652 | 0.601967944 |
| EM | 2.577275 | 7.522595 | 2.856247 | 5.062556 | -0.034998289 | 0.491251321 |
| EM | 2.615288 | 7.586537 | 4.476671 | 5.322887 | 1.257261239 | 0.778554285 |
| EM | 2.642208 | 7.712123 | 3.79923 | 4.733058 | 0.970496324 | 0.725218415 |
| EM | 2.646945 | 7.72595 | 2.702908 | 5.211299 | -0.383236709 | 0.40534648 |
| EM | 2.65431 | 7.747447 | 2.710429 | 4.47092 | 0.154387214 | 0.538520321 |
| EM | 2.557942 | 7.079197 | 2.985768 | 4.8597 | 0.778930199 | 0.685449501 |
| EM | 2.913979 | 6.650353 | 2.574228 | 5.162572 | 1.644991245 | 0.838212953 |
| EM | 2.555488 | 7.38483 | 2.887701 | 5.557231 | -0.237135515 | 0.440992378 |
| EM | 2.509666 | 7.325259 | 2.562727 | 5.330387 | -0.4007926 | 0.401121924 |
| EM | 2.894292 | 6.87916 | 2.989718 | 5.117331 | 1.699559186 | 0.845477153 |
| EM | 3.018061 | 6.56669 | 2.658417 | 5.146461 | 2.104663221 | 0.891355594 |
| EM | 3.617909 | 6.301717 | 2.675603 | 4.332002 | 4.563878513 | 0.989685928 |
| EM | 4.629985 | 6.94029 | 3.643892 | 4.914009 | 6.678233523 | 0.998743582 |
| EM | 2.482686 | 7.053797 | 2.51504 | 5.12831 | 0.001290988 | 0.500322747 |
| EM | 2.992465 | 6.954965 | 2.607219 | 5.954832 | 0.881565994 | 0.707146628 |
| EM | 2.822708 | 7.142213 | 2.621251 | 4.81068 | 1.057049239 | 0.742126247 |
| EM | 2.688281 | 7.145032 | 3.285455 | 5.432872 | 0.868152671 | 0.704361162 |
| EM | 2.569382 | 7.279918 | 2.546865 | 5.223224 | -0.127403979 | 0.468192019 |
| EM | 2.76856 | 6.199162 | 4.376989 | 4.416008 | 4.075370911 | 0.983297789 |
| EM | 3.6316 | 6.194498 | 2.641446 | 4.559751 | 4.544423902 | 0.989485438 |
| EM | 2.445622 | 7.321314 | 2.497329 | 4.695132 | -0.149784276 | 0.462623784 |
| EM | 2.852717 | 6.97596 | 2.616455 | 4.963321 | 1.23914157 | 0.775414557 |
| EM | 2.985993 | 6.83639 | 3.104179 | 4.905749 | 2.243943032 | 0.904126791 |
| EM | 2.468178 | 6.974043 | 2.519264 | 4.841117 | 0.286218848 | 0.571070193 |
| EM | 3.130493 | 6.878637 | 2.569718 | 4.616109 | 2.27219231 | 0.906547684 |
| EM | 2.582559 | 7.335339 | 3.893417 | 4.781002 | 1.379105835 | 0.798847355 |
| EM | 2.50246 | 7.686034 | 2.555369 | 4.996773 | -0.666313113 | 0.339322887 |
| EM | 3.472887 | 6.800149 | 4.061181 | 4.784804 | 4.462627351 | 0.988599447 |
| EM | 3.644577 | 6.625206 | 2.598884 | 4.720763 | 3.841436747 | 0.978988228 |
| EM | 2.484245 | 7.251059 | 2.665248 | 5.400403 | -0.322243279 | 0.420129141 |
| EM | 3.495835 | 7.068529 | 3.187578 | 5.275567 | 3.005538225 | 0.952823699 |
| EM | 2.911873 | 6.938795 | 2.545965 | 4.623389 | 1.620655312 | 0.834885486 |
| EM | 2.600941 | 6.516314 | 2.708246 | 5.005259 | 1.28345175 | 0.783036768 |
| EM | 2.580348 | 7.75503 | 2.683498 | 5.193809 | -0.592827598 | 0.355986335 |
| EM | 3.442483 | 6.610944 | 2.671141 | 4.807941 | 3.359929187 | 0.966428479 |
| EM | 3.490003 | 6.432276 | 2.625774 | 4.892645 | 3.615029698 | 0.973789363 |
| EM | 3.64584 | 6.40967 | 2.927328 | 4.955696 | 4.261216588 | 0.986091056 |
| EM | 3.884835 | 6.818298 | 4.335251 | 5.224193 | 5.390883699 | 0.995462737 |
| EM | 2.872992 | 6.573879 | 2.542396 | 4.879159 | 1.824089691 | 0.861056134 |
| EM | 2.758638 | 6.496018 | 2.586051 | 6.415378 | 0.560749663 | 0.63662598 |
| EM | 2.499701 | 7.263449 | 2.552551 | 4.964513 | -0.08424814 | 0.478950414 |
| EM | 2.429077 | 7.109775 | 2.470144 | 4.729359 | 0.04359215 | 0.510896312 |
| EM | 2.641003 | 6.600278 | 2.829039 | 4.83822 | 1.502272172 | 0.817913118 |
| EM | 2.423808 | 7.245399 | 2.517686 | 4.722237 | -0.103321978 | 0.47419246 |
| EM | 2.376718 | 7.747323 | 2.282083 | 4.699625 | -1.092749951 | 0.251100798 |
| EM | 2.521376 | 6.344631 | 2.557337 | 4.966224 | 1.207544645 | 0.769864214 |
| EM | 2.580084 | 6.856478 | 2.571641 | 4.878723 | 0.742696939 | 0.677585321 |
| EM | 2.49257 | 7.201005 | 2.490931 | 5.203583 | -0.248923388 | 0.438088508 |
| EM | 2.383633 | 7.21922 | 2.52563 | 5.075195 | -0.418916975 | 0.396775938 |
| EM | 2.419569 | 7.062281 | 2.470725 | 5.042443 | -0.144624012 | 0.463906886 |
| EM | 2.418849 | 7.147046 | 2.45073 | 4.706843 | -0.033131392 | 0.49171791 |
| EM | 2.49548 | 6.512267 | 2.557769 | 4.672577 | 1.132955488 | 0.756383911 |
| EM | 2.590398 | 6.793245 | 2.500876 | 4.365493 | 1.164223245 | 0.762099253 |
| EM | 2.478919 | 7.133162 | 2.204965 | 3.457579 | 0.824821938 | 0.695258944 |
| EM | 2.404673 | 7.141532 | 2.486727 | 4.159604 | 0.371896011 | 0.591917042 |
| EM | 2.265863 | 7.986716 | 2.518471 | 5.441234 | -2.01741519 | 0.11738653 |
| EM | 2.363197 | 7.354033 | 2.523529 | 4.670533 | -0.35694735 | 0.411698727 |
| EM | 2.458983 | 7.53388 | 2.187232 | 4.474559 | -0.523010023 | 0.372148659 |
| EM | 2.389137 | 7.619922 | 2.210068 | 5.627126 | -1.63433061 | 0.163237974 |
| EM | 2.394301 | 7.957812 | 2.237251 | 5.10361 | -1.66805624 | 0.158683505 |
| EM | 2.499386 | 7.833866 | 2.400524 | 4.643682 | -0.755304753 | 0.319666522 |
| EM | 2.496332 | 8.366171 | 2.329589 | 5.057255 | -1.845176571 | 0.136440218 |
| EM | 2.455033 | 7.320796 | 2.192528 | 4.443432 | -0.218898794 | 0.445492778 |

Note: linear_predictors = expression value of SSTR5*2.48 - expression value of CASP3*1.34 + expression value of FABP2*0.9 - expression value of SYK*0.73 + 4.76;

The risk score represents the probability value that the multifactor model predicts for each sample.
